# C-C Bonding in Molecular Systems via Cross-Coupling-like Reactions Involving Noncovalently Bound Constituent Ions

**DOI:** 10.3390/molecules29184429

**Published:** 2024-09-18

**Authors:** Stephen Kerr, Fedor Y. Naumkin

**Affiliations:** Faculty of Science, Ontario Tech University/UOIT, Oshawa, ON L1G 0C5, Canada; stephen.kerr1@ontariotechu.net

**Keywords:** intermolecular complexes, ion pairs, cross-coupling reactions, ab initio calculations

## Abstract

Carbon-based molecules are of universal importance for a huge variety of chemical and biological processes. The complication of the structure of such molecules proceeds via the bonding of carbon atoms. An efficient mechanism for such reactions proceeds via cross-coupling, related to the association of bond-terminating counter-ions. Here, an uncommon version of such a process is investigated, with at least some ions bound in the system noncovalently and/or switching the bonding mode in due course. The analyzed sample reactions involve a single C-C bond formation in environmentally relevant halocarbon species and involve alkali–halide ion-pair components. A consistent ab initio computational study predicts the related energy barriers to alter significantly in the presence of the ion pair. Different channels are checked, with the carbon–halogen bond cleavage preceding or following the actual C-C bonding and with the counter-ions located closely or farther apart. The relative heights of the corresponding energy barriers are found to be switched by the ion pair. The above results suggest a possibility of facilitating such reactions without expensive catalysts.

## 1. Introduction

The formation of C-C bonds is a central process in chemistry (e.g., with regard to polymerization, film and crystal growth, and various other ways of developing complex structures) and biology (in particular with relevance for the growth of living organisms). It is therefore difficult to overestimate its importance and the need to better understand its features and versatility.

One of the efficient mechanisms (celebrated by a Noble Prize award [1]) of forming C-C bonds is cross-coupling, which proceeds via the close approach of, e.g., halogen (X) and metal (M) atoms terminating bonds originating on different C atoms. This can lead to the M-X associating into a metal–halide diatom, accompanied by coupling the released C bonds and binding these C atoms. The purpose of the present work is to evaluate the feasibility of such a process for the associating atoms attached to the carbon bases noncovalently, especially not next to each other.

One realistic version of such a situation is the case when one of the atoms, e.g., X, is initially attached covalently, and the reaction leads to its transition to noncovalent attachment before the association with M occurs. This can be exemplified by the recently studied M-CF_3_ + CF_4_ → C_2_F_6_ + MF cross-coupling-like reaction [2] proceeding via the transformation of CF_4_ into CF_3_-F with one unbonded F (to form, in particular, a metastable intermediate M-C_2_F_6_-F complex), further associating with an alkali metal atom M. The latter atom, in turn, can be already attached noncovalently in the reactant species (here, M-CF_3_).

Ion pairs noncovalently attached to molecules may not necessarily result in bond formation via a cross-coupling or (e.g., in case of halo-organic rather than organic species) a cross-coupling-like reaction. In other cases, they could cause a reshaping of a trapped molecule, such as the unfolding of its bent structure under the pressure of mutually attracting counter-ions [3,4] or a molecule’s isomerization via bond rearrangement, as in M-BzO_3_-X → M-C_6_O_3_H_6_ [5] and M-cubane-X → M-ladderene-X [6]. Additionally, an ion-pair-framed molecule could also preserve its integrity and overall shape while the system demonstrates notable modifications of its other properties. These can include polarity (with outstanding dipoles up to dozens of Debye), microwave and IR spectra (with considerably increased intensities), etc., as in M-C_n_H_n_F_n_-X (n = 3–6) [4,7,8,9,10], M-Bz-X [11,12,13,14], and M-C_n_H_2n_-X (n = 3, 6) [8,15,16]. In the latter case, the inserted nonpolar molecule (e.g., a hydrocarbon) stretches the ion pair, resulting in metastability of the system, while the polar insert (such as an all-*cis* halo-hydrocarbon) can stabilize the system relative to the separate molecule and ion pair, and even make it more stable than the isomeric complex of the directly attached components, as in *molecule*-MX. MX-trapped larger molecules (or, in other words, molecules such as the receptors of both M and X) have also been investigated [17], including, for instance, cyclic species such as hexacyclen and calixpyrrole [18,19]. In particular, cyclic inserts have appropriate concave electron densities (lower at centers) accommodating the framing ions and preventing their association around the trapped molecules, especially the nonpolar ones.

In particular, alkali-metal and halogen atoms represent a typical suitable pair in cross-coupling reactions due to their strong ionic bonding. The present work investigates a cross-coupling-like reaction M-CCl_3_ + CCl_4_ → C_2_Cl_6_ + MCl more completely than its previously studied F-based counterpart [2], including a variety of M-CCl_3_ reactant conformers and following a couple of possible reaction channels. Significant differences in the alteration in the relevant potential barrier are found as compared to the previous case.

It is worth noting that CCl_4_ has currently been phased out because of concerns about its environmental impact (precursor to refrigerants depleting ozone in the atmosphere) and safety (negative effects on the nervous system, liver, and kidneys) [20]. C_2_Cl_6_ has been used for extreme-pressure lubricants as well as in veterinary practice and for treatments against fungi and insects [21]. So, reacting CCl_4_ into C_2_Cl_6_ transforms a harmful substance into a useful one, and improving the process efficiency would be beneficial.

## 2. Results and Discussion

First, the halocarbon system itself is considered. Next, an alkali metal atom is added in different ways, and the resulting alterations in the parameters of interest are analyzed.

### 2.1. CCl_3_-CCl_4_ → C_2_Cl_6_-Cl

**Figure 1:**
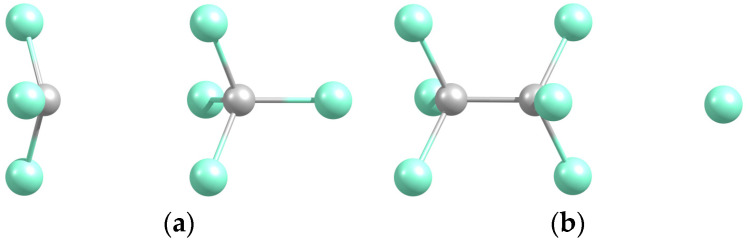
Optimized geometries of the CCl_3_-CCl_4_ (**a**) and C_2_Cl_6_-Cl (**b**) complexes.

We begin with the reactant and product systems, then follow their transformation. The optimized Cl_3_-CCl_4_ structure has the components in a staggered (in terms of the CCl bonds) arrangement (Figure 1), while C_2_Cl_6_-Cl corresponds to the atom positioned axially relative to the molecule. Both complexes are weakly bound (Table 1), the former being more stable due to the dipole-induced dipole interaction.

**Figure 2:**
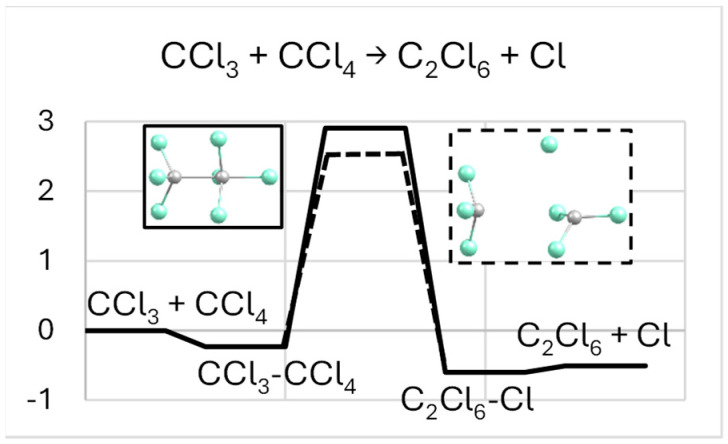
Energy diagram (in eV) for the CCl_3_ + CCl_4_ → C_2_Cl_6_ + Cl reaction. The reactions can be led by a C-C bond forming (solid line) or a C-Cl bond breaking (dashed), as described in the text, with the corresponding transition states shown in matching frames.

Shrinking the C-C distance in CCl_3_-CCl_4_ while reoptimizing the rest of the atomic coordinates presses the two molecules axially into one another, inverting the CCl_3_ part of CCl_4_ (like an umbrella in a strong wind) and detaching its axial Cl atom (positioned at the system axis). This leads to C-C bonding and C_2_Cl_6_-Cl forming over a barrier of about 3 eV (Figure 2). In comparison, for the analogous reaction CF_3_-CF_4_ → C_2_F_6_-F, the barrier was predicted to be somewhat lower, about 2.4 eV [2]. A similar transformation can also be achieved via stretching the radial (facing the CCl_3_ molecule) C-Cl bond in CCl_4_, which causes the CCl_3_ remainder to axially align and merge with the CCl_3_ molecule into C_2_Cl_6_, followed by about equally weak sideways attachment of the withdrawn Cl to it. The corresponding energy barrier is found to be about 0.5 eV lower.

### 2.2. NaCCl_3_

Here, we start with NaCCl_3_ complexes, then proceed to their interactions with CCl_4_.

#### 2.2.1. Structures and Stabilities

**Figure 3:**
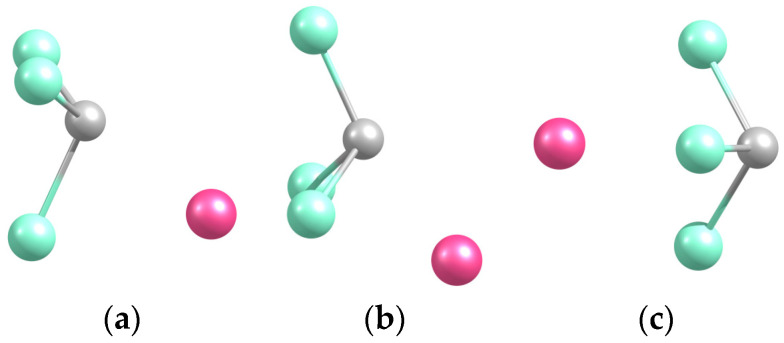
Optimized geometries of the Na-CCl_3_ (**a**), Na-Cl_2_CCl (**b**), and Na-Cl_3_C (**c**) complexes.

Three conformers were predicted, with Na in front of a CCl edge, a CCl_2_ face (the structure is denoted NaCl_2_CCl based on the proximity of atoms), and the Cl_3_ base (NaCl_3_C) (Figure 3). The three structures are close in energy, within 0.1 eV, the first one being the least and the second one being the most bound (Table 2). The latter correlates with the shortest Na-C distance (C being the most negatively charged, as discussed below) and with the proximity of Na to two Cl atoms. The near-equal stability of NaCCl_3_ and NaCl_3_C can also be correlated to an interplay between the relative Na-C separation (shorter in the former) and the number of Cl atoms in proximity to Na (larger in the latter). The analogous NaCF_3_ system exhibits similar features to its conformers [2].

**Figure 4:**
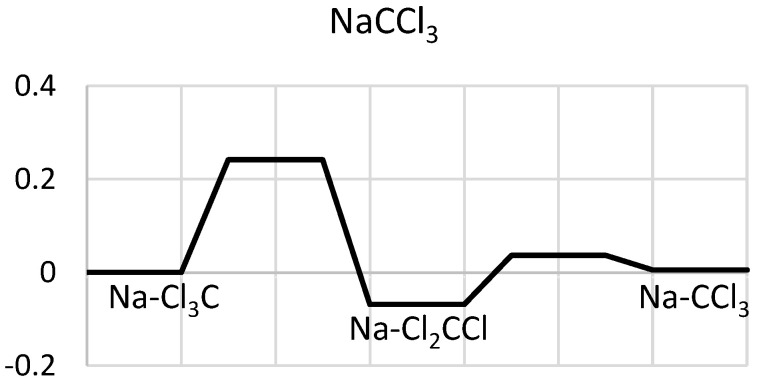
Energy diagrams (in eV) for the conformations of NaCCl_3_.

The NaCl_3_C species is separated by a one-quarter eV barrier from NaCl_2_CCl, and NaCCl_3_ is near-degenerate with NaCl_3_C (Figure 4), with a tiny energy barrier (under 0.05 eV) towards NaCl_2_CCl. The intuitive axial position of Na in front of C corresponds to a saddle point.

#### 2.2.2. Charge Distributions

For all conformers, the Na atom expectedly transfers electron density (near-unit charge) to CCl_3_ (Table 3). The Cl atoms closer to Na are more negative, and the C atom is also less negative in Na-Cl_3_C, where it is farther from Na.

Such charge distributions correlate to the “collective” electrostatic bonding introduced for such systems recently [22] and are here associated with a few negative centers (C and Cl atoms). Apparently, the degree of collectivity varies among the three conformers, ranging from two (C and one Cl) to four (C and three Cls) major anionic contributors.

#### 2.2.3. Simulated IR Spectra

**Figure 5:**
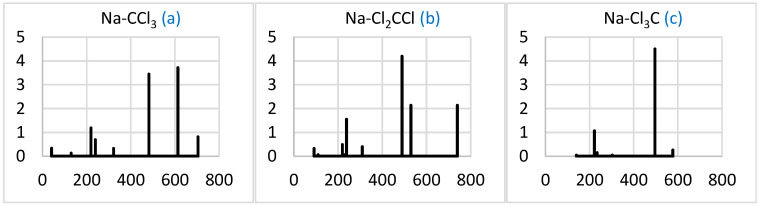
Simulated IR spectra (intensity in (D/Å)^2^ vs. frequency in cm^–1^) of the Na-CCl_3_ (**a**), Na-Cl_2_CCl (**b**), and Na-Cl_3_C (**c**) complexes. Letters of systems correspond to those in Figure 3. The spectral data can be found in Table S1 (in the Supplementary Materials).

The predicted IR intensity distribution is sensitive to the Na-CCl_3_ conformation (Figure 5). The spectra are mainly concentrated around 500 cm^–1^. The most symmetric Na-Cl_3_C is dominated by a single band splitting in two (about 130 cm^–1^ apart) in Na-CCl_3_ and (to a lesser extent) Na-Cl_2_CCl. The latter conformer, however, also develops a significant higher-frequency band near 740 cm^–1^. The weaker band near 200 cm^–1^ is common for the three conformers.

### 2.3. NaCCl_3_-CCl_4_ → C_2_Cl_6_-NaCl

Next, each of the above NaCCl_3_ conformers is complexed with a CCl_4_ molecule.

#### 2.3.1. Structures and Stabilities

**Figure 6:**
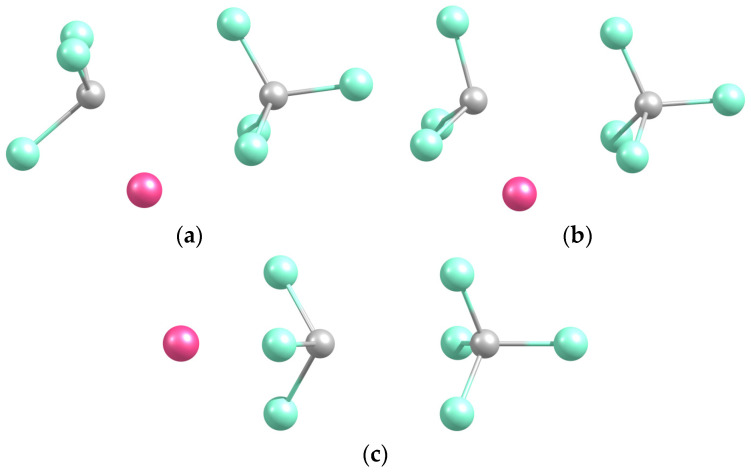
Optimized geometries of the Na-CCl_3_-CCl_4_ (**a**), Na-Cl_2_CCl-CCl_4_ (**b**), and Na-Cl_3_C-CCl_4_ (**c**) complexes.

The optimized structures have the CCl_3_ units of NaCCl_3_ oriented similarly to those of Cl_3_-CCl_4_ (Figure 6), with slight distortions due to the Na components. In fact, these systems can also be produced from Cl_3_-CCl_4_ by attaching Na sideways or axially. In particular, Na-CCl_3_-CCl_4_ somewhat stabilizes the Na facing the CCl edge of CCl_3_ due to this position now being in the hollow among three Cl atoms, while Na-Cl_2_CCl-CCl_4_ aligns CCl_3_ and CCl_4_ in terms of the C-Cl bonds via making a square hollow for Na among four Cl atoms. As a result, the C-C distance slightly stretches in those cases but slightly shrinks in Na-Cl_3_C-CCl_4_ (Table 4) by about 0.2 Å in both cases.

The two complexes with Na on a side are near-equally stable, while the one with axially positioned Na is about half as stable (Table 4), consistent with the weaker interaction of Na with the more remote CCl_4_. The higher stabilization for Na-CCl_3_-CCl_4_ than for Na-Cl_2_CCl-CCl_4_ (inverting their relative stability compared to that of Na-CCl_3_ vs. Na-Cl_2_CCl) could be related to strain in the latter due to the abovementioned Na-caused relative rotation of the CCl_3_ and CCl_4_ units from the staggered to the aligned arrangement.

**Figure 7:**
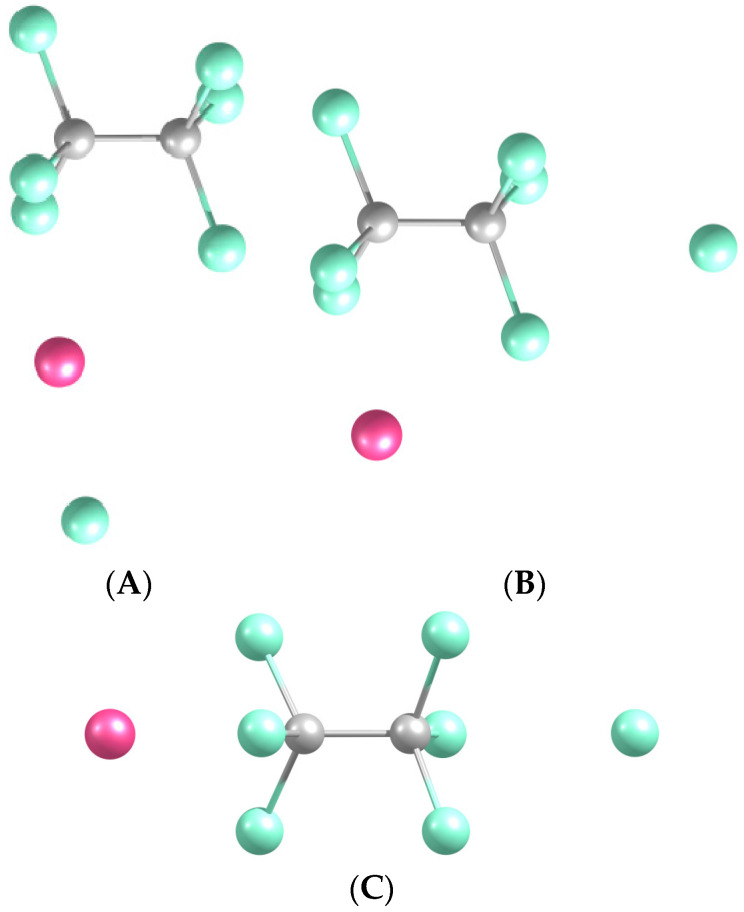
Optimized geometries of the NaCl-C_2_Cl_6_ (**A**), Na-C_2_Cl_6_-Cl (L) (**B**), and Na-C_2_Cl_6_-Cl (**C**) complexes. Here, L denotes the L-shaped case.

Shrinking the C-C bond leads to the formation of C_2_Cl_6_, with the axially positioned Cl of CCl_4_ detaching, similar to the case when no Na is present. For the original Na-Cl_2_CCl-CCl_4_ and Na-Cl_3_C-CCl_4_, the resulting geometries again resemble those obtained via the attachment of Na to the C_2_Cl_6_-Cl system perpendicular to or along its axis (Figure 7), respectively, producing Na-C_2_Cl_6_-Cl (L-shaped) and Na-C_2_Cl_6_-Cl complexes. The former has the Na atom attached to the side of C_2_Cl_6_ but with a tiny potential barrier separating the Na and axial Cl from an association around C_2_Cl_6_ into NaCl and with the formation of a C_2_Cl_6_-NaCl system, with NaCl attached sideways and pointing to C_2_Cl_6_ from its Na end. The latter system is also a direct product of the case of the corresponding Na-CCl_3_-CCl_4_ transformation.

Essentially, the Na–Cl distance (hence, charge separation in the ion pair) determines the relative stabilities of the three above structures (Table 4), from moderately stable (by a half eV), electrostatically bound C_2_Cl_6_-NaCl, to metastable Na-C_2_Cl_6_-Cl (both conformers). The Cl atom here is the one not bonded to the C atoms and accepts the electron density from Na (see Section 2.3.2 below). And, the metastability means a higher energy relative to C_2_Cl_6_ + NaCl (in this case, due to the far-separated counter-ions), hence a negative D_e_ value.

**Figure 8:**
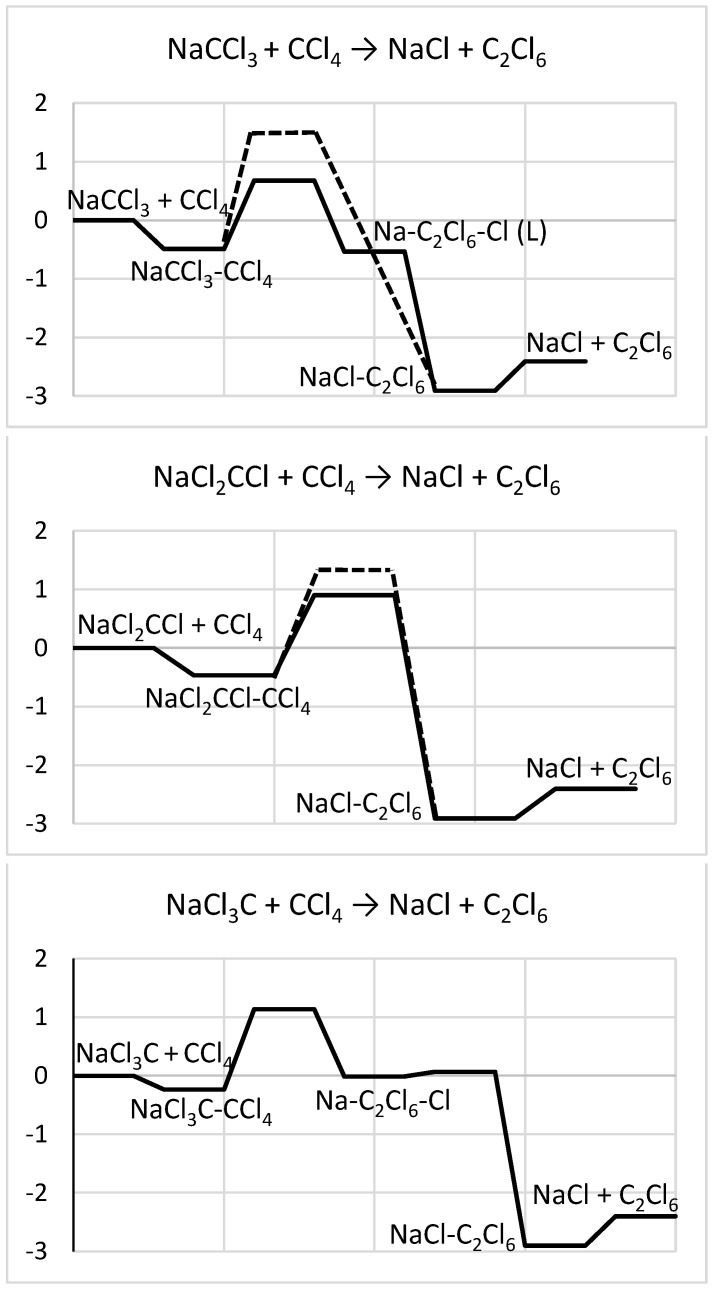
Energy diagrams (in eV) for the reactions (top to bottom) NaCCl_3_ + CCl_4_ → NaCl + C_2_Cl_6_, NaCl_2_CCl + CCl_4_ → NaCl + C_2_Cl_6_, NaCl_3_C + CCl_4_ → NaCl + C_2_Cl_6_. (L) denotes the L-shaped case. The reactions can be led by C-C bond forming (solid line) or C-Cl bond breaking (dashed line).

The original (reactant) and resulting (product) species are again separated by a potential barrier (Figure 8), similar to the case without Na. However, the presence of Na strongly reduces its height (about three–fourfold), progressively from Na-Cl_3_C + CCl_4_ (about 1.1 eV) to Na-CCl_3_ + CCl_4_ (about 0.7 eV). Such a barrier suppression could be assigned to the attraction between the Na and axial (released in the process) Cl, increasing with decreasing Na–Cl distance in this order of conformers. In addition, the Na-C_2_Cl_6_-Cl complex with the molecule axially trapped between the counter-ions shows a very low potential barrier (under 0.1 eV) to their association (around the molecule), leading to the sideways-attached NaCl-C_2_Cl_6_ system. In comparison, the corresponding barrier for the similar F-based case (only the Na-F_3_C-CF_4_ system considered) reduces weakly, to 1.7 eV [2], and is determined by a metastable Na-C_2_F_7_ species (not having a Cl-based counterpart) slightly lower in energy than Na-C_2_F_6_-F.

The alternative channel via C-Cl bond stretching also exhibits considerable potential-barrier reductions for Na-CCl_3_ + CCl_4_ and Na-Cl_2_CCl + CCl_4_, even though smaller (about twofold, to 1.4–1.5 eV). In particular, when Cl is pulled away in the latter case, the structure of the former system is recovered, and the process then follows the same steps. As a result of the different variations, the relative heights of the two barriers interchange as compared to those of the no-Na case. For Na-Cl_3_C + CCl_4_, however, the detaching Cl atom of CCl_4_ is overtaken by the CCl_3_ component, thus forming another CCl_4_, which blocks the formation of C_2_Cl_6_. A possible reason for the latter could be that the Na in the axial position pulls Cl towards the C of CCl_3_, favoring new C-Cl bond formation (unlike for the other cases with Na positioned off-axis and pulling Cl sideways from the C of CCl_3_). As a result, an isomeric Na-CCl_4_-CCl_3_ system is produced, with Na on the CCl_4_ side. The potential barrier here is about 3.2 eV.

#### 2.3.2. Charge Distributions

In all the Na-containing systems studied here, Na is positively charged by almost unity. In the “reactant” species, the electron density is transferred to the CCl_3_ component, with the (closed-shell) CCl_4_ molecule remaining almost neutral (Table 5). And, in the “products”, the charge concentrates on the unbonded Cl atom (Table 6), while C_2_Cl_6_ is essentially neutral, even when it is positioned between Na and Cl, i.e., directly in the way of the charge transfer. The Na-C_2_Cl_6_-Cl complex may thus be considered a result of “harpooning” through the trapped molecule.

#### 2.3.3. Simulated IR Spectra

**Figure 9:**
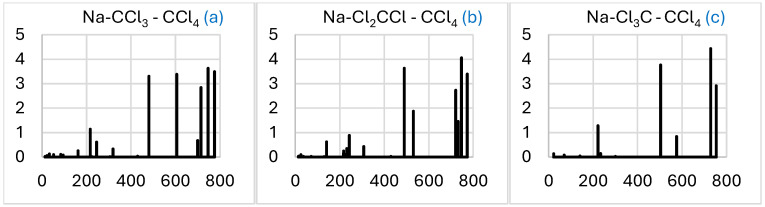
Simulated IR spectra (intensity in (D/Å)^2^ vs. frequency in cm^–1^) of the Na-CCl_3_-CCl_4_ (**a**), Na-Cl_2_CCl-CCl_4_ (**b**), and Na-Cl_3_C-CCl_4_ (**c**) complexes. Letters of systems correspond to those in Figure 6. The spectral data can be found in Table S2.

The comparison of the simulated IR spectra for the three Na-CCl_3_-CCl_4_ conformers with those for the corresponding Na-CCl_3_ (Figure 5) shows a common feature—a few intense closely packed higher-frequency bands in the range of 700–800 cm^–1^ (Figure 9). The number of different bands is smaller for Na-Cl_3_C-CCl_4_ due to the higher symmetry of the system, and their origin is apparently the CCl_4_ molecule, with its main near-800 cm^–1^ band red-shifted due to the interaction with Na-CCl_3_.

**Figure 10:**
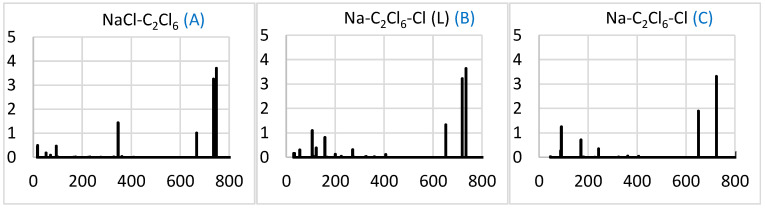
Simulated IR spectra (intensity in (D/Å)^2^ vs. frequency in cm^–1^) of the NaCl-C_2_Cl_6_ (**A**), Na-C_2_Cl_6_-Cl (L) (**B**), and Na-C_2_Cl_6_-Cl (**C**) complexes. Here, (L) denotes the L-shaped case. Letters of systems correspond to those in Figure 7. The spectral data can be found in Table S3.

The IR spectra of the three products are mainly similar, being dominated by the band matching the near-750 cm^–1^ band of C_2_Cl_6_ (Figure 10). Again, this band is split in NaCl-C_2_Cl_6_ and (more appreciably) Na-C_2_Cl_6_-Cl (L), due to the interaction with the other components, but not in the more symmetric Na-C_2_Cl_6_-Cl. Another notable modification of the spectra in the complexes is a set of additional less-intense bands at low frequencies, mainly under 400 cm^–1^ for NaCl-C_2_Cl_6_ and under 200 cm^–1^ for both Na-C_2_Cl_6_-Cl conformers.

A comparison of the spectra in Figure 9 and Figure 10 shows that the main variation is the cancellation of the intense bands in the range of about 450–600 cm^–1^, apparently associated with the CCl_3_ component. This is consistent with the consumption of this component in the reaction that enables its spectroscopic control.

### 2.4. CsCCl_3_-CCl_4_ → C_2_Cl_6_-CsCl

Here, a comparison with the analogous Cs-based systems is briefly highlighted. The heavier Na-CCl_3_ counterpart, Cs-CCl_3_, exhibits very similar near-degenerate CCl_3_-Cs and Cs-Cl_2_CCl conformers, the latter again being slightly more stable. However, a structure with Cs bridging C and one Cl is not found. These species are about 0.5 eV more strongly bound than the Na-based counterparts, likely due to a stronger charge transfer from the less-electronegative Cs and its higher polarizability.

The corresponding complexes with CCl_4_ are bound about equally for CCl_3_-Cs and Cs-Cl_2_CCl and comparably to the Na-based analogues. The corresponding Na-Cl_2_CCl-CCl_4_ complex is slightly more bound (by about 0.2 eV), perhaps due to the smaller Na being closer to CCl_4_ (which is less significant for Na-Cl_3_C-CCl_4_ in view of the larger Na-CCl_4_ separation).

The potential energy barriers for the formation of C_2_Cl_6_ via C-C bond shrinking in Cs-Cl_3_C-CCl_4_ and Cs-Cl_2_CCl-CCl_4_ are about 0.9 and 1.0 eV, respectively. These are about 0.2 eV lower or 0.1 eV higher than those for the Na-based analogues and about 0.5 eV lower than for the corresponding fluorocarbon system [2]. The final product is the same in either case, C_2_Cl_6_-CsCl, with an intermediate metastable Cs-C_2_Cl_6_-Cl system (stabilized by even lower potential barriers than in Na-C_2_Cl_6_-Cl) for the former channel. For C-Cl bond stretching, the potential barriers are about 3.2 eV for Cs-Cl_3_C-CCl_4_ and 2.1 eV for Cs-Cl_2_CCl-CCl_4_. These values are, respectively, the same as for Na-Cl_3_C-CCl_4_ and about 0.6 eV higher than for Na-Cl_2_CCl-CCl_4_. Again, similar to the Na-based case, for the former channel, the formation of C_2_Cl_6_ is blocked, while the latter channel leads to C_2_Cl_6_-CsCl.

## 3. Computational Methods

In the present work, the studied molecular systems involve both covalent and noncovalent interactions between their fragments. To consistently deal with both such components, a reasonable combination of sufficient accuracy and affordable computation time is offered by the Moller–Plessett perturbation theory of the 2nd order (MP2). Here, the appropriate aug-cc-pVTZ basis set for C and Na, and the relativistic effective core potentials (Stuttgart RLC ECP) [23] for Cl and Cs were selected. The above theoretical approach was employed via the ab initio program package NWChem [24].

The tests included the most relevant interactions in the system, C-Cl and Na-Cl. Specifically, the dissociation energies and equilibrium distances for CCl_4_ and NaCl were calculated, leading to D_e_(Cl-CCl_3_) = 3.507 eV at R_e_ = 1.735 Å and D_e_(Na-Cl) = 4.235 eV at R_e_ = 2.378 Å, favorably comparing to the respective experimental values of 3.074 eV at 1.767 Å and 4.272 ± 0.087 eV at 2.361 Å. Additionally, the dipole moment of NaCl was calculated as 9.25 D, closely matching the 9.00 D from the experiments.

The computational procedure involved full optimizations of the system geometries and confirmations of energy minima in terms of vibrational frequency analyses. The transition states, if found instead, were dealt with using the associated eigenvectors.

The IR intensity spectra were also produced based on NWChem calculations within the harmonic approximation. In particular, test comparisons of the predictions at this level and the available experimental [25] spectra for CCl_4_ and C_2_Cl_6_ show very close matches in the band frequencies and relative intensities.

The atomic charges were evaluated using the natural population analysis (NPA) [26]. It was employed via JANPA software (version 2.02) [27].

## 4. Conclusions

A C-C bond-forming reaction involving small chlorocarbons was considered at a consistent MP2 level of theory with and without an alkali metal added. Three near-degenerate conformers of Na-CCl_3_ species were employed, differing in the position of Na relative to CCl_3_. Each conformer makes a distinct corresponding conformer of the reactant complex Na-CCl_3_-CCl_4_. Upon reaction, the final product complex is C_2_Cl_6_-NaCl for most cases, with possible intermediate systems, including uncommon Na-C_2_Cl_6_-Cl with the ion-pair-trapped molecule. In particular, the latter and similar cases involve the “umbrella” inversion of the CCl_3_ unit, while, in other cases, it is just reoriented.

Two possible channels of the reaction can be followed, with either the C-C distance shortened or the C-Cl bond stretched (followed by C-C bonding). In the absence of Na, the corresponding potential barriers are comparable, the one for the former channel being about a half eV higher. Upon adding Na, the potential barriers are considerably reduced for both channels, more so for direct C-C bonding (by an impressive factor of 3–4). This also interchanges the relative heights of the barriers, making the C-Cl bond-stretch-related channel less likely. In particular, such a potential barrier reduction is much stronger compared to that of the corresponding fluorocarbon system.

The effect is apparently due to the formation of a Na–Cl ion-pair, which involves the Cl atom detaching from CCl_4_ and subsequently associating with Na. C-Cl bond stretching or C-C bond shrinking releases Cl, respectively, close to Na, which corresponds to a cross-coupling-like process, or distant from Na, via an intermediate structure with this Cl atom noncovalently bound as well. The alkali metal thus appears to be an inexpensive promoter of the process, which usually needs costly catalysts such as Pd.

In particular, CCl_3_ can be produced via the photolysis of CCl_4_, while atomic Na could likely be obtained by laser vaporization. Na-CCl_3_ complexes could possibly be formed experimentally in crossed beams of these components or by the photolysis of tetrachloromethane in presence of sodium vapor. The latter option might even facilitate the complete reaction under study here, perhaps then to be compared with a similar process in the absence of Na to test the predictions.

The simulated IR spectra are sensitive to the system structure and facilitate the experimental identification of the relevant species. The spectral variation allows tracking the reaction progress as well.

## Figures and Tables

**Table 1:** Equilibrium parameters (dissociation energies, distances) of the studied systems.

System	D_e_/eV	R_e_(C-C)	R_e_(C-Cl *)
CCl_3_-CCl_4_ (a)	0.231	3.586	1.736
C_2_Cl_6_-Cl (b)	0.095	1.554	3.743

* Axial Cl atom (positioned at the system axis). Letters with systems correspond to those in Figure 1.

**Table 2:** Equilibrium parameters (dissociation energies, distances) of binary systems.

System	D_e_/eV	R_e_(Na-C)/Å	R_e_(Na-Cl *)
Na-CCl_3_ (a)	2.334	2.297	2.638
Na-Cl_2_CCl (b)	2.408	2.225	2.697
Na-Cl_3_C (c)	2.339	2.831	2.613

* Nearest Cl atom(s). Letters of systems correspond to those in Figure 3.

**Table 3:** Natural atomic charges in the binary systems.

System	q(Na)/e	q(C)	q(Cl)
Na-CCl_3_	0.956	−0.549	−0.080, −0.247
Na-Cl_2_CCl	0.956	−0.520	−0.036, −0.200
Na-Cl_3_C	0.918	−0.302	−0.205

**Table 4:** Equilibrium parameters (dissociation energies, distances) of the ternary systems.

System	D_e_/eV	R_e_(Na-C ^#^)/Å	R_e_(C-C)	R_e_(C-Cl *)	R_e_(Na-Cl ^&^)
Na-CCl_3_-CCl_4_ (a)	0.488 ^†^	2.286	3.797	1.729	2.635
Na-Cl_2_CCl-CCl_4_ (b)	0.465 ^†^	2.231	3.756	1.730	2.680
Na-Cl_3_C-CCl_4_ (c)	0.235 ^†^	2.825	3.349	1.745	2.615
NaCl-C_2_Cl_6_ (A)	0.502 ^‡^	3.309	1.555		2.384
Na-C_2_Cl_6_-Cl (L ^$^) (B)	−1.869 ^‡^	3.101	1.557	3.334	2.579
Na-C_2_Cl_6_-Cl (C)	−2.391 ^‡^	2.828	1.549	3.275	2.732

**^#^** Nearest C atom. * Axial Cl atom (positioned at the system axis). ^&^ Nearest Cl atom(s). ^†^ Relative to binary complex + CCl_4_. ^‡^ Relative to NaCl + C_2_Cl_6_. ^$^ L-shaped. Letters of systems correspond to those in Figure 6 and Figure 7.

**Table 5:** Natural atomic charges in the reactant systems.

System	q(Na)/e	q(CCl_3_)	q(CCl_4_)
Na-CCl_3_-CCl_4_ (a)	0.927	−0.957	0.030
Na-Cl_2_CCl-CCl_4_ (b)	0.920	−0.950	0.030
Na-Cl_3_C-CCl_4_ (c)	0.919	−0.929	0.010

Letters of systems correspond to those in Figure 6.

**Table 6:** Natural atomic charges in the product systems.

System	q(Na)/e	q(Cl)	q(C_2_Cl_6_)
NaCl-C_2_Cl_6_ (A)	0.914	−0.951	0.037
Na-C_2_Cl_6_-Cl (L ^$^) (B)	0.949	−0.983	0.034
Na-C_2_Cl_6_-Cl (C)	0.954	−0.984	0.030

^$^ L-shaped. Letters of systems correspond to those in Figure 7.

## Data Availability

The data presented in this study are available upon request from the corresponding author.

## Supplementary Materials

The following supporting information can be downloaded at: https://www.mdpi.com/article/10.3390/molecules29184429/s1; Table S1: Calculated IR spectra parameters for the Na-CCl_3_ conformers; Table S2: Calculated IR spectra parameters for the Na-CCl_3_-CCl_4_ conformers; Table S3: Calculated IR spectra parameters for the NaCl-C_2_Cl_6_ conformers.
