# Supplementary material for: C-C Bonding in Molecular Systems via Cross-Coupling-like Reactions Involving Noncovalently Bound Constituent Ions
Source: Molecules. 2024 Sep 18;29(18):4429. doi: 10.3390/molecules29184429 (PMC11433999; doi:10.3390/molecules29184429)
Supplement: Supplementary file 1 [file molecules-29-04429-s001.zip › molecules-3163549-supplementary.pdf]

C-C bonding in molecular systems via cross-coupling-like reactions involving non-covalently bound constituent ions

Stephen Kerr [1], Fedor Y. Naumkin [1,*]

[1] Faculty of Science, Ontario Tech University / UOIT, Oshawa, ON, L1G 0C5, Canada
[*] Correspondence: Fedor.Naumkin@uoit.ca

Table S1. Calculated IR spectra parameters for the Na-CCl_3 conformers (Figure 5)

<u>Na-CCl_3</u>

Frequency /cm^{-1}	Intensity / $(D/Å)^2$
41.562	0.340
129.947	0.130
219.428	0.011
220.093	1.186
239.482	0.695
322.119	0.323
482.121	3.446
613.245	3.710
704.620	0.818

<u>Na-Cl_2CCl</u>

Frequency /cm^{-1}	Intensity / $(D/Å)^2$
90.936	0.330
109.293	0.062
219.023	0.493
231.489	0.063
238.364	1.552
309.341	0.398
489.683	4.196
530.171	2.135
739.809	2.137

Na-Cl_3C

Frequency /cm^{-1}	Intensity / (D/Å)^2
141.070	0.048
141.081	0.048
221.491	1.073
234.583	0.154
234.627	0.154
302.645	0.049
495.864	4.505
495.884	4.505
576.851	0.257

Table S2. Calculated IR spectra parameters for the Na-CCl_3-CCl_4 conformers (Figure 9)

Na-CCl_3-CCl_4

Frequency /cm^{-1}	Intensity / (D/Å)^2
13.587	0.022
22.374	0.064
32.474	0.121
40.073	0.002
51.918	0.100
83.586	0.110
94.513	0.084
161.847	0.258
216.465	0.007
217.411	1.136
218.346	0.002
223.880	0.016
245.486	0.607
303.868	0.010

305.575	0.012
311.651	0.004
319.149	0.328
428.990	0.030
480.600	3.293
605.435	3.382
699.838	0.674
714.899	2.841
746.357	3.627
776.078	3.493

$Na-Cl_2CCl-CCl_4$

Frequency /cm^{-1}	Intensity / (D/Å)^2
14.146	0.030
17.538	0.023
25.344	0.101
36.906	0.022
70.974	0.027
82.616	0.000
129.502	0.019
140.452	0.618
216.607	0.150
217.473	0.242
224.542	0.094
231.276	0.345
242.090	0.883
304.598	0.008
305.338	0.037
308.054	0.431
311.896	0.011

429.333	0.022
490.102	3.629
530.909	1.867
720.932	2.732
731.975	1.455
747.544	4.054
772.681	3.386

$Na\text{-}Cl_3C\text{-}CCl_4$

Frequency /cm^{-1}	Intensity / (D/Å)^2
22.701	0.019
22.933	0.133
23.216	0.135
47.974	0.001
70.007	0.078
70.097	0.078
141.077	0.040
141.106	0.040
218.980	0.001
219.049	0.000
221.841	1.281
233.688	0.142
233.713	0.142
299.666	0.029
306.799	0.001
306.818	0.001
307.544	0.001
430.969	0.002
502.891	3.759
502.914	3.760

575.039	0.834
728.318	4.430
753.682	2.913
753.691	2.913

Table S3. Calculated IR spectra parameters for the NaCl-C_2Cl_6 conformers (Figure 10)

NaCl-C_2Cl_6

Frequency /cm^{-1}	Intensity / (D/Å)^2
16.738	0.443
17.333	0.483
52.142	0.182
70.177	0.086
93.668	0.459
100.027	0.016
164.894	0.004
173.263	0.016
224.597	0.001
226.013	0.000
231.996	0.011
270.721	0.000
275.839	0.002
326.550	0.002
329.411	0.017
345.935	1.439
361.977	0.034
409.674	0.007
666.689	1.011
735.809	3.254
747.774	3.698

820.885	0.071
836.388	0.064
1064.535	0.005

Na-C_2Cl_6-Cl (L)

Frequency /cm^{-1}	Intensity / $(D/Å)^2$
30.420	0.158
33.008	0.162
55.492	0.300
105.753	1.095
109.544	0.050
122.265	0.379
157.983	0.805
167.271	0.008
200.198	0.128
225.178	0.037
227.660	0.002
268.183	0.000
271.601	0.307
277.850	0.006
323.711	0.025
326.940	0.031
360.542	0.024
407.161	0.113
652.272	1.334
719.418	3.226
734.545	3.635
820.464	0.251
827.262	0.180
1041.290	0.031

Na-C_2Cl_6-Cl

Frequency /cm^{-1}	Intensity / $(D/Å)^2$
46.604	0.023
46.726	0.022
87.964	0.272
88.287	0.272
91.532	1.248
94.837	0.000
171.142	0.710
181.571	0.017
181.679	0.017
227.736	0.006
227.798	0.006
242.775	0.347
269.107	0.000
269.150	0.000
324.178	0.014
324.197	0.014
361.411	0.052
406.630	0.039
650.748	1.891
724.332	3.308
724.473	3.310
806.640	0.199
806.751	0.195
1048.589	0.046
